# Trends in Mortality From Aortic Stenosis in Europe: 2000–2017

**DOI:** 10.3389/fcvm.2021.748137

**Published:** 2021-10-11

**Authors:** Adam Hartley, Matthew Hammond-Haley, Dominic C. Marshall, Justin D. Salciccioli, Iqbal S. Malik, Ramzi Y. Khamis, Joseph Shalhoub

**Affiliations:** ^1^National Heart and Lung Institute, Imperial College London, London, United Kingdom; ^2^Imperial College Healthcare NHS Trust, London, United Kingdom; ^3^Department of Cardiology, Hammersmith Hospital, London, United Kingdom; ^4^British Heart Foundation Centre of Excellence, King's College London, London, United Kingdom; ^5^Department of Respiratory Medicine, St Mary's Hospital, London, United Kingdom; ^6^Pulmonary and Critical Care Medicine, Brigham and Women's Hospital and Harvard Medical School, Boston, MA, United States; ^7^Academic Section of Vascular Surgery, Department of Surgery and Cancer, Imperial College London, London, United Kingdom; ^8^Department of Vascular Surgery, St Mary's Hospital, London, United Kingdom

**Keywords:** aortic stenosis, mortality, Europe, transcatheter aortic valve implantation (TAVI), aortic valve replacement (AVR)

## Abstract

**Background:** Trends in mortality from aortic stenosis across European countries are not well-understood, especially given the significant growth in transcatheter aortic valve implantation (TAVI) in the last 10 years.

**Methods:** Age-standardised death rates were extracted from the World Health Organisation Mortality Database, using the International Classification of Diseases 10th edition code for non-rheumatic aortic stenosis for those aged > 45 years between 2000 and 2017. The UK and countries from the European Union with at least 1,000,000 inhabitants and at least 50% available datapoints over the study period were included: a total of 23 countries. Trends were described using Joinpoint regression analysis.

**Results:** No reductions in mortality were demonstrated across all countries 2000–2017. Large increases in mortality were found for Croatia, Poland and Slovakia for both sexes (>300% change). Mortality plateaued in Germany from 2008 in females and 2012 in males, whilst mortality in the Netherlands declined for both sexes from 2007. Mortality differences between the sexes were observed, with greater mortality for males than females across most countries.

**Conclusions:** Mortality from aortic stenosis has increased across Europe from 2000 to 2017. There are, however, sizable differences in mortality trends between Eastern and Western European countries. The need for health resource planning strategies to specifically target AS, particularly given the expected increase with ageing populations, is highlighted.

## Introduction

Aortic stenosis (AS) is the most prevalent valvular heart disease in Europe (1). Although rheumatic heart disease is the most common aetiology worldwide, degenerative calcification of native trileaflet valves and congenital bicuspid valves are the primary causes in developed countries. AS prevalence increases significantly with advancing age and is ~10% over the age of 80 (2). Moreover, with ageing populations, the burden is expected to increase further (3).

AS prognosis relates predominantly to severity and symptoms, with over 50% mortality at 1-year with conservative treatment for severe symptomatic AS (4). Although traditionally the outlook for asymptomatic AS has been considered favourable, frequently managed with routine surveillance, this has been brought into focus in a recent meta-analysis which demonstrated significant cardiovascular mortality in this patient group (5).

Until relatively recently, the only treatment available for AS that provided prognostic benefit was surgical aortic valve replacement (SAVR). However, the first transcatheter aortic valve implantation (TAVI) was performed in 2002 (6), offering potential intervention to those who were previously denied invasive treatment based on advancing age or high operative risk, with a dramatic improvement in mortality in these patients (7). Widespread uptake of TAVI has subsequently been seen, with excellent outcomes and increasing procedural scope, and is now even indicated in patients with low surgical risk (8). Indeed, across Western Europe, TAVI procedures have increased almost 350% from 2010 to 2019 (9). Yet access to TAVI is not universal, and there is significant regional variation, strongly related to healthcare resource availability (10). A recent analysis of mortality in the USA demonstrated declining mortality trends in older patients with AS (in line with increased TAVI procedures), which was not seen in patients from non-metropolitan areas (11).

We sought to explore changing trends in AS mortality across Europe between 2000 and 2017, given the introduction, exponential uptake and expanding indications of TAVI. This will aim to demonstrate changing patterns of mortality with access to modern therapies and highlight regional healthcare inequalities that require targeted intervention.

## Methods

### Data Sources

Data were extracted from the World Health Organisation (WHO) Mortality Database for the years 2000–2017. Data quality is continuously assessed by the WHO to ensure reliability and usability. Birth and population recording must exceed 90% for countries to be included in the database. Details of data collection and validation for the database have been described extensively previously (12, 13). The WHO Mortality Database uses International Classification of Disease (ICD) codes to classify causes of death. The tenth revision ICD code for non-rheumatic AS (I35.0) was used, whilst ICD codes pertaining to aortic insufficiency or mixed aortic valve disease were excluded.

Member states of the European Union (EU) as well as the United Kingdom, with populations > 1,000,000, were included in the study. EU countries Malta, Cyprus and Luxembourg were not included owing to having <1,000,000 inhabitants. Greece and Ireland were excluded due to more than 50% missing datapoints for the study period. All included countries had high quality cause of death data, except Bulgaria, Poland and Portugal (medium quality). Data are considered high quality if the country reports at least 5 years of data, uses ICD codes for at least the latest year, and has data usability (as defined by the WHO) of at least 80%. Data are considered medium quality using the same criteria as high quality, but with average data usability of at least 60% (12).

Therefore, 23 countries were included in the final analysis. The estimated level of completeness of death registration reported to the WHO Mortality Database for all included countries was at least 97% up to 2010, and 100% for the most recent year, up to 2016 (14). Death rates were computed from vital registration data for all included countries, with the exception of Lithuania which was based on annual estimates informed by completeness-adjusted vital registration data and United Nations population assessments (12). Mortality data were further restricted to those above the age of 45 years, to limit the effects of more severe congenital disease, and focus on a generalisable population that may be suitable for conventional medical and surgical interventions. This age restriction has been utilised previously (11). Not all countries had data available for 2017, and as such, four countries (Belgium, Estonia, Italy, and UK) reported up to 2016, three (Bulgaria, Denmark, and Latvia) reported up to 2015 and two (France and Slovakia) reported up to 2014.

### Data Handling

Crude annual national mortality data with annual national population data were extracted from the WHO Mortality Database. Following this, the age standardised death rate (ASDR) was calculated. This relates the distribution of mortality per 5-year age group per country, weighted according to the WHO standard population (15). This standardisation process is commonly used in mortality epidemiological studies and controls for differences in age structure, permitting more robust comparison between countries. In this study we report ASDR per 1,000,000 population, as performed previously (11). The data underlying this article are available in the article and can be found at https://www.who.int/data/data-collection-tools/who-mortality-database. Patients or the public were not involved in the design or conduct of this study. Ethical approval was not required for this study, given that the data is collected from widely available, internationally collected mortality certification.

### Statistical Analysis

Sex-specific trends for AS age-standardised mortality were established and analysed for 3-year periods at the start and end of the observation period. Where 3-year data were missing, at either the start or end of the observation period, the 2-year or 1-year averages were calculated, as appropriate. Where no data were available for any timepoint for either of these periods (2000–2002 or 2015–2017), the earliest and latest available timepoints were used. Percentage change was assessed between the earliest and latest values.

Mortality trends were assessed using Joinpoint Version 4.5.0.1 Command (US National Cancer Institute Surveillance Research Program). For the purpose of Joinpoint analysis, missing data were imputed in a last observation carried forward method. Joinpoint regression analysis assesses changes in linear gradients for ASDR over time, as performed previously (16). In brief, Joinpoint analysis initially assesses the overall trends in mortality without any joinpoints, and then evaluates for changes in the model, with the addition of further joinpoints for each statistically significant slope change. A log-linear transformation is performed, permitting approximation to normal distributions and each additional joinpoint is tested for significance using a Monte Carlo permutation method. Estimated annual percentage change (EAPC) for each trend is calculated by fitting a regression line to the natural logarithm of the rates. Each EAPC is assessed to determine whether a significant difference exists compared to no change in mortality. The final model consists of multiple joinpoints, each representing a significant change in trend, with each trend described by EAPC and confidence intervals. A statistically significant difference was defined as a two-sided *p*-value < 0.05.

Differences in the change in ASDR per sex between newer EU joining countries and older countries from 2000 to 2017 were assessed with the Mann-Whitney test. EU-joining nations from 2004 or later were deemed new, as has been used previously (16).

## Results

Over the study period between 2000 and 2017, there were significant changes in AS mortality across European countries in those aged > 45 years, for both males and females. Twenty-three countries from the EU and UK were included in the final analysis, after exclusion of countries with significant missing data or with <1,000,000 inhabitants. The quality of the mortality data in the WHO Mortality Database has been reviewed previously to ensure sufficient reliability and robustness (13). In total, mortality data were missing for 8.7% of all potential values during the observation period.

### Overall Changes in Mortality From Aortic Stenosis

Between 2000 and 2017 there were increases in mortality from AS in all countries, for both sexes. The differences in ASDRs between the start and end of the study period are shown in Table 1. Very large increases in mortality were reported in Croatia, Czech Republic, Estonia, Poland, Slovakia, and Slovenia for both sexes (>100% change). Small increases in mortality were reported for the majority of countries, including Belgium, Denmark, Finland, Hungary, Italy and UK (15–50% change for both sexes). The Netherlands was the only country that reported no substantial changes (<5% change for both sexes).

**Table 1:** Change in age-standardised death rates from non-rheumatic aortic stenosis for those aged > 45 years in Europe from 2000 to 2017.

**Country**	**Start**		**End**		**Raw change**		**% change**	
	**Male**	**Female**	**Male**	**Female**	**Male**	**Female**	**Male**	**Female**
Austria	17.64	15.58	27.76	26.56	10.12	10.98	57.4	70.48
Belgium	19.49	19.49	22.43	24.15	2.94	4.66	15.06	23.93
Bulgaria^*^	3.05	1.31	3.11	2.83	0.06	1.52	2.12	115.51
Croatia	5.69	1.97	33.81	26.86	28.12	24.89	493.93	1,265.62
Czech Republic	7.68	4.29	24.25	16.25	16.57	11.97	215.87	279.26
Denmark	19.03	18.89	28.09	24.99	9.06	6.1	47.62	32.28
Estonia	13.09	5.07	30.49	18.08	17.4	13.01	132.97	256.58
Finland	30.87	24.45	43.03	31.86	12.16	7.41	39.4	30.32
France^†^	17.97	13.68	20.81	15.69	2.85	2	15.84	14.63
Germany	15.55	14.5	29.61	26.53	14.06	12.03	90.47	82.97
Hungary	22.35	14.92	28.63	21.83	6.29	6.91	28.13	46.35
Italy	7.83	8.03	9.94	12.02	2.11	3.99	26.95	49.72
Latvia	7.92	4.39	8.61	9.42	0.69	5.02	8.7	114.45
Lithuania	7.09	1.34	8.86	6.25	1.77	4.91	24.95	367.28
Netherlands	21.66	20.45	21.82	20.92	0.16	0.47	0.75	2.3
Poland	3.42	1.72	14.79	11.08	11.37	9.36	333.02	545.1
Portugal^€^	12.21	11.51	19.57	20.52	7.36	9.01	60.28	78.32
Romania	3.2	2.19	4.68	4.18	1.47	1.99	46.01	90.91
Slovakia^§^	3.82	0.85	17.04	14.41	13.22	13.55	346.19	1,587.77
Slovenia	34.7	26.12	71.35	63.8	36.65	37.68	105.64	144.24
Spain	10.87	12.5	22.55	19.46	11.68	6.96	107.44	55.68
Sweden	23.2	19.09	25.79	20.48	2.59	1.39	11.18	7.28
United Kingdom	18.86	13.8	26.18	19.07	7.32	5.27	38.82	38.15

*Average estimated annual percentage change (EAPC) between 2000–2002 and 2015–2017 used, where data available. The following countries did not have available EAPC for one of those periods, and therefore the following intervals were used:*

* *Bulgaria 2005–2015;*

† *France 2000–2014;*

€ *Portugal 2007–2017;*

§ *Slovakia 2000–2014*.

**Figure 1:**
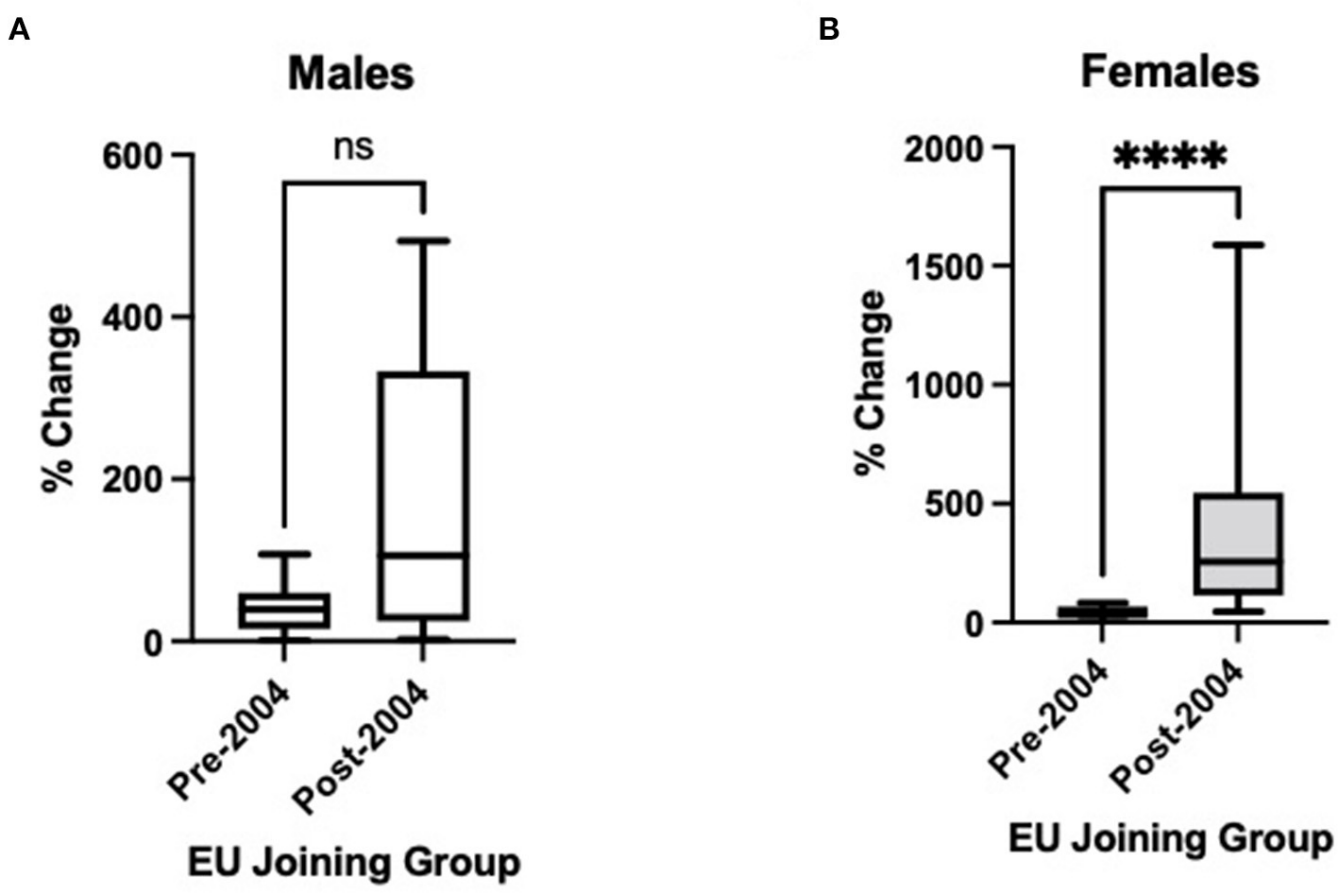
Changes in age-standardised death certification rates per 1,000,000 for countries joining the European Union (EU) before 2004 and 2004 or later, 2000 to 2017, for non-rheumatic aortic stenosis for those aged > 45 years in **(A)** males and **(B)** females. Pre-2004 countries: Austria, Belgium, Denmark, Finland, France, Germany, Italy, Netherlands, Portugal, Spain, Sweden, and the United Kingdom. Post-2004 countries: Bulgaria, Croatia, Czech Republic, Estonia, Hungary, Latvia, Lithuania, Poland, Romania, Slovakia, and Slovenia. Rather than 2000–2017, the following intervals are used owing to data availability: Bulgaria 2005–2015; France 2000–2014; Portugal 2007–2017; Slovakia 2000–2014. Mann-Whitney test used to assess statistical significance. ns, non-significant (*p* = 0.17); ****, statistical significance at *p* < 0.0001.

Figure 1 displays the percentage change in ASDR 2000–2017 for both males (A) and females (B) from Table 1, divided into earlier (pre-2004) and later (post-2004) EU joining nations. Although there is an apparent trend only for males (*p* = 0.17), there is a strongly statistically significant difference for females (*p* < 0.0001), with much greater increases in ASDR for later EU joining countries.

### Joinpoint Regression Analysis of Mortality From Aortic Stenosis

**Figure 2:**
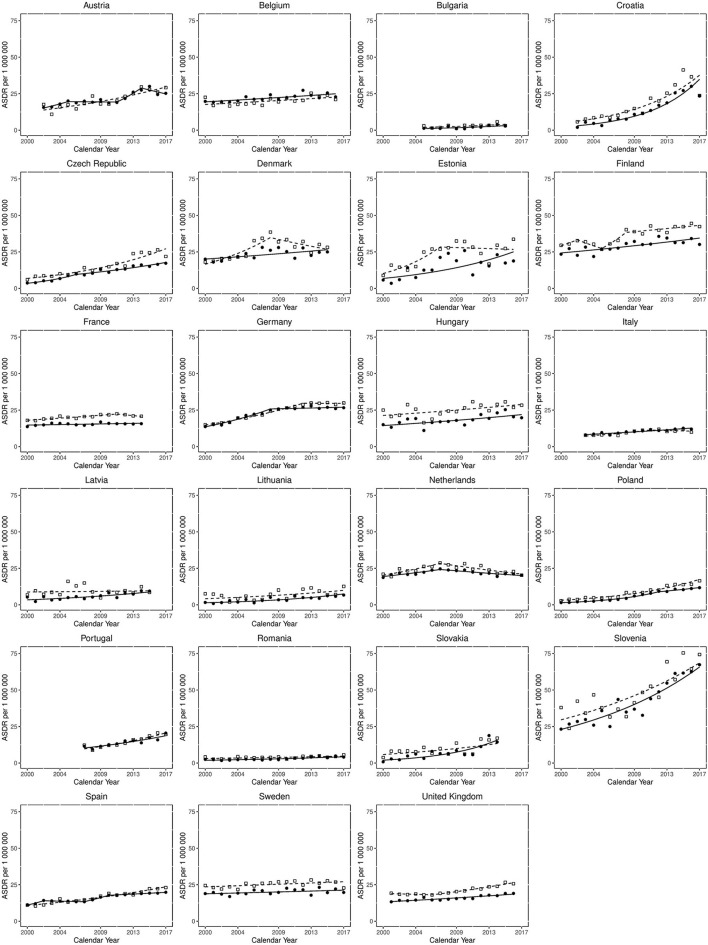
Joinpoint regression analysis for trends in age-standardised death rates from non-rheumatic aortic stenosis for those aged > 45 years in Europe from 2000 to 2017. Clear squares indicate males; filled circles indicate females. The lines (dotted for males, solid for females) represent modelled trends based on joinpoint data.

Figure 2 displays the results of Joinpoint regression analysis for trends in mortality for each country per sex. The most common trend was for a slow sustained increased ASDR over the study period, as can be seen in Belgium, Hungary, Italy, Lithuania, Spain and the UK, for both sexes, and Latvia and Denmark for females. Steeper increases in mortality over the study period are observed in both sexes in the Czech Republic, Slovakia, Poland and Portugal, as well as Estonia for females and Austria for males. However, the steepest increases are seen in Croatia and Slovenia for both sexes. Of note, very low levels of mortality are reported in Bulgaria and Romania, with ASDRs <10 per 1,000,000 population throughout the observation period. The death rates in Sweden and France for both sexes, as well as Latvia for males, were relatively static.

Despite there being no mortality reductions from the start to end of the study period, some improving mortality trends are observed. Mortality plateaued in Germany from 2008 in females and 2012 in males, whilst in the Netherlands mortality declined for both sexes from 2007. For males in Denmark mortality declined from 2008 and there was also a small decline in Estonian males from 2007. Lastly, there was a reduction in mortality for Austrian females from 2014.

Mortality differences between the sexes were also observed, with consistently greater mortality for males than females in a large proportion of countries. This sex gap appears to be narrowing in some countries (for example Denmark, Estonia and Latvia), but not all (for example Czech Republic and UK, where the difference is growing), and is stable in most.

### Estimated Annual Percentage Change in Mortality From Aortic Stenosis

The EAPCs, shown in Table 2, highlight the differing trends between Eastern and Western European countries. Mortality in both sexes is consistently and statistically significantly increasing with an EAPC of >5 in Croatia, Czech Republic, Lithuania, Poland, Portugal, Slovakia and Slovenia. For males in Austria, as well as females in Estonia and Latvia, statistically significant increasing trends are also seen. The largest single trend increasing EAPCs in males are seen in Croatia {12.8 [95% confidence interval (95% CI) +10.2 to −15.5]}, Poland [+10.6 (95% CI +9.4 to −11.8)] and Czech Republic [+8.5 (95% CI +7.2 to −9.8)]. The largest single trend EAPCs in females are observed in Croatia [+17.6 [95% CI +14.3 to −21.0)], Slovakia [+15.9 (95% CI +9.9 to −22.4)] and Bulgaria [+10.8 [95% CI +2.4 to −19.9)].

**Table 2:** Joinpoint regression analysis for mortality from non-rheumatic aortic stenosis for those aged > 45 years in Europe from 2000 to 2017.

**Country**	**Sex**	**Trend 1**		**Trend 2**		**Trend 3**		**Trend 4**	
		**Years**	**EAPC**	**Years**	**EAPC**	**Years**	**EAPC**	**Years**	**EAPC**
Austria	Males	2002–2017	5.2 (3.4–7.0)^*^						
	Females	2002–2005	8.8 (−3.0 to 22.1)	2005–2011	−0.2 (−5.2 to 5.0)	2011–2014	14.4 (−9.1 to 44.0)	2014–2017	−4.9 (−15.2 to 6.7)
Belgium	Males	2000–2016	1.6 (0.5–2.7)^*^						
	Females	2000–2016	1.6 (0.9–2.3)^*^						
Bulgaria	Males	2005–2015	7.8 (−0.6 to 16.8)						
	Females	2005–2015	10.8 (2.4–19.9)^*^						
Croatia	Males	2002–2017	12.8 (10.2–15.5)^*^						
	Females	2002–2017	17.6 (14.3–21.0)^*^						
Czech	Males	2000–2017	8.5 (7.2–9.8)^*^						
Republic	Females	2000–2006	18.3 (13.5–23.3)^*^	2006–2017	5.8 (4.0–7.5)^*^				
Denmark	Males	2000–2008	9.8 (5.8–13.9)^*^	2008–2015	−3.7 (−8.0 to 0.8)				
	Females	2000–2015	1.9 (0.5–3.3)^*^						
Estonia	Males	2000–2007	15.7 (5.5–26.9)^*^	2007–2016	−0.7 (−6.7 to 5.8)				
	Females	2000–2016	8.5 (3.7–13.4)^*^						
Finland	Males	2000–2002	6.7 (−8.5 to 24.6)	2002–2005	−6.4 (−19.8 to 9.3)	2005–2008	12.0 (−4.0 to 30.7)	2008–2017	1.3 (−0.1 to 2.8)
	Females	2000–2017	2.1 (1.2–3.0)^*^						
France	Males	2000–2011	2.0 (1.3–2.7)^*^	2011–2014	−2.5 (−7.3 to 2.6)				
	Females	2000–2014	0.6 (0.0–1.2)^*^						
Germany	Males	2000–2012	6.0 (5.4–6.6)^*^	2012–2017	−0.1 (−2.1 to 2.0)				
	Females	2000–2008	8.4 (7.0–9.8)^*^	2008–2017	0.5 (−0.6 to 1.6)				
Hungary	Males	2000–2017	1.8 (0.4–3.2)^*^						
	Females	2000–2017	2.5 (1.0–4.0)^*^						
Italy	Males	2003–2011	5.9 (2.8–9.1)^*^	2011–2016	−1.4 (−7.3 to 4.8)				
	Females	2003–2016	3.4 (2.5–4.3)^*^						
Latvia	Males	2000–2015	0.6 (−3.1 to 4.4)						
	Females	2000–2015	6.5 (3.4–9.7)^*^						
Lithuania	Males	2000–2017	5.2 (1.2–9.4)^*^						
	Females	2000–2017	10.3 (7.3–13.5)^*^						
Netherlands	Males	2000–2007	4.9 (2.2–7.7)^*^	2007–2017	−2.9 (−4.4 to −1.4)^*^				
	Females	2000–2007	3.2 (1.5–5.0)^*^	2007–2017	−1.9 (−2.8 to −0.9)^*^				
Poland	Males	2000–2017	10.6 (9.4–11.8)^*^						
	Females	2000–2012	16.6 (14.9–18.3)^*^	2012–2017	5.7 (−0.1 to 11.8)				
Portugal	Males	2007–2017	7.8 (5.3–10.3)^*^						
	Females	2007–2017	6.2 (4.0–8.4)^*^						
Romania	Males	2000–2017	2.5 (0.7–4.3)^*^						
	Females	2000–2017	5.0 (3.4–6.6)^*^						
Slovakia	Males	2000–2014	6.1 (1.3–11.0)^*^						
	Females	2000–2014	15.9 (9.9–22.4)^*^						
Slovenia	Males	2000–2017	5.1 (3.2–7.0)^*^						
	Females	2000–2017	6.4 (5.0–7.7)^*^						
Spain	Males	2000–2017	4.7 (4.0–5.3)^*^						
	Females	2000–2002	12.7 (7.9–17.7)^*^	2002–2007	−0.9 (−2.3 to 0.4)	2007–2010	10.0 (5.3–14.8)^*^	2010–2017	1.5 (1.0–2.1)^*^
Sweden	Males	2000–2017	0.8 (0.1–1.5)^*^						
	Females	2000–2017	0.7 (0.0–1.5)						
United	Males	2001–2006	−0.8 (−2.5 to 0.8)	2006–2016	3.8 (3.2–4.4)^*^				
Kingdom	Females	2001–2016	2.2 (1.6–2.7)^*^						

*Where data are available. EAPC, estimated annual percentage change*.

* *Statistical significance, p < 0.05*.

Although consistent declining trends are seen Estonia, France, Italy and Netherlands for males, Netherlands and Austria are the only countries with declining female mortality. Indeed, Netherlands is the only country with any statistically significant decreasing trend for any period across the study, with EAPC of −2.9 (95% CI −4.4 to −1.4) and −1.9 (95% CI −2.8 to −0.9) for males and females, respectively.

## Discussion

This study of deaths from AS between 2000 and 2017 identifies increases in mortality in all European countries. There are however substantially differing trends between countries, with some reporting stable ASDRs across the observation period, some reporting changing trends towards reduction in mortality, whilst others report rapidly worsening mortality rates. Mortality from AS is shown to increase significantly in many Eastern European countries, for example Croatia, Czech Republic, Poland, Slovakia and Slovenia for both sexes. This divide is evident when examining earlier and later EU-joining nations (Figure 1), particularly in females. Of note, Estonia has the smallest population of all included countries, which may explain the more variable datapoints. In addition, the low level of mortality from AS in Bulgaria and Romania may represent under-reporting, perhaps due to a lower AS diagnosis rate. However, many Western European countries also demonstrate increasing trends, most notably Portugal. Germany and Netherlands are the only countries that have plateauing or declining mortality rates for both sexes, and Netherlands is the only country where the decreasing trends were statistically significant. Nonetheless, increases in mortality over the whole study period are observed in all countries; possibly related to population ageing or an increased likelihood of AS diagnosis with greater access to diagnostics.

The only countries with plateauing or declining mortality for both sexes (Germany and Netherlands) were early TAVI adopters, and have well-established TAVI practise and registries. Indeed, Germany performs the most TAVIs in Europe, whilst TAVI far exceeds SAVR in the Netherlands (17, 18). Procedural complications and mortality reduce with greater TAVI experience (19), which may contribute, along with the greater TAVI numbers, to the declining AS mortality in these countries. Conversely, Croatia had only performed 87 TAVIs by 2014 (20), Slovenia was a slow adopter and had only performed procedures in one centre in 2015 (21) and Poland has among the lowest numbers of TAVI procedures in Europe (22). Portugal, a Western European country with increasing AS mortality, had the lowest number of TAVI procedures in Europe (7 per 1,000,000 population/year) in 2013 (23). More recently, in a survey of TAVI practise from 20 European countries for 2018, Slovenia (6 per million inhabitants) and Poland (22 per million inhabitants) had the lowest reported figures (Croatia was not included in the study), whilst Germany had the highest (187 per million inhabitants). Portugal performed only 30 per million inhabitants (24).

However, the link between developments in TAVI practise and trends in mortality from AS cannot be assumed to be causal. For example, a possible explanation is that TAVI uptake could be a surrogate for other indicators of good clinical practise. Moreover, TAVI and SAVR rates are not collected uniformly, and are gained from multiple sources. There are also other potential factors that influence AS mortality. For example, experience and practise of SAVR, frequency of AS diagnosis, as well as the prevalence and management of AS risk factors (e.g., chronic renal disease, hypercholesterolaemia, diabetes, and smoking) (25). There is significant overlap between these risk factors and those for atherosclerotic cardiovascular disease. Of note, we have previously demonstrated growing disparity in mortality from cardiovascular disease between Western and Eastern European countries (16).

An interesting observation from the present study is the difference in mortality between males and females that, for the majority of countries, is not narrowing. This sex disparity contradicts the current belief that females have worse outcomes from AS. Theories behind this include that females are more likely to be older and frailer at presentation; have higher pulmonary pressures and more concomitant mitral valve disease (26); and that AS may be more common in females, related to longer life expectancy as well as smaller aortic root dimensions (27). However, males have a much higher preponderance of cardiovascular risk factors (which are mostly shared with AS) and have a lower left ventricular ejection fraction at presentation (28). Females present later into the disease course with more insidious symptoms; thus AS incidence, and therefore mortality, may be under-represented in this group (26). Procedural factors suggest more inherent risks during TAVI on female patients, for example smaller cardiac structures increasing technical complexity and more difficult vascular access (29). Despite this, TAVI outcomes are actually superior in females and indeed may be the preferred treatment modality in older women (30).

Challenges to wider adoption of TAVI in less economically developed countries include the availability of resources, education, infrastructure and diagnostic services. TAVI procedures are expensive, due to high device costs, necessary infrastructure including cardiothoracic surgical services, and the need for dedicated multi-disciplinary Heart teams. Any increase in TAVI use has to be justified in terms of both survival and quality of life benefits.

The morbidity and mortality related to AS in Europe and North America is likely to be very different to that of lower income countries. This relates to aetiology, where degenerative rather than infectious causes (infective endocarditis or rheumatic disease) predominate, the population affected (elderly vs. younger), as well as the healthcare resources available to detect and treat the condition. Although no worldwide comparisons of AS mortality have been performed, mortality from rheumatic heart diseases does appear to be decreasing (31).

Limitations of this study include the reliability of large scale retrospective data; however accuracy is assessed by the WHO, and there is at least medium quality data reporting and at least 98% death coverage for all included countries in this analysis (12). Nonetheless, data coding may be of differing quality between countries and this may impact on the observed results. There is also the potential for missed mortality trends, with absent datapoints for some countries. In addition, there may be a significant diagnosis bias in reporting of mortality. For example, AS will be detected at a greater rate with more widespread access to echocardiography, and therefore will feature more commonly on death certification. It is possible that more developed healthcare systems have more established echocardiography services. Another potential factor is the improving quality of contemporary cardiac ultrasound machines; AS may be detected more frequently on images with greater spatial and temporal resolution. As stated above, a causal link between mortality rates and TAVI use cannot be inferred from this study. The effect of underdiagnosis on the treatment of AS unfortunately cannot be assessed in this study, as the WHO collect data on mortality with no information available on reported prevalence.

## Conclusion

Mortality from aortic stenosis has increased across Europe between 2000 and 2017. There are, however, sizable differences in mortality trends between Eastern and Western European countries. Significant plateauing or declining mortality is observed for recent years in countries with greater access to TAVI, whilst increasing trends are observed in countries with the least TAVI use. The need for health resource planning strategies to specifically target AS, due to the expected increase in incidence with ageing populations, is highlighted.

## Data Availability Statement

The raw data supporting the conclusions of this article are freely available from the World Health Organisation (WHO) Mortality Database.

## Author Contributions

JS, JDS, DM, and AH designed the study. JDS and DM performed data analysis. AH wrote the manuscript, which was reviewed and edited by MH-H, JS, JDS, DM, IM, and RK. AH is the guarantor of the manuscript. All authors contributed to the article and approved the submitted version.

## Funding

This work was supported by a Wellcome Trust Clinical Research Fellowship (220572/Z/20/Z) (AH).

## Conflict of Interest

The authors declare that the research was conducted in the absence of any commercial or financial relationships that could be construed as a potential conflict of interest. The reviewer FB-L declared a shared affiliation, with one of the authors JDS to the handling editor at the time of the review.

## Publisher's Note

All claims expressed in this article are solely those of the authors and do not necessarily represent those of their affiliated organizations, or those of the publisher, the editors and the reviewers. Any product that may be evaluated in this article, or claim that may be made by its manufacturer, is not guaranteed or endorsed by the publisher.
